# Uncovering hidden HCV infections in adults over 55: reflex testing and insights for opportunistic screening in a tertiary care setting

**DOI:** 10.1186/s13690-026-01866-7

**Published:** 2026-03-18

**Authors:** Emma Montella, Rita Nocerino, Simona Del Sorbo, Stefano Brusa, Giuseppe Jannuzzi, Biagio Pinchera, Valentina Cossiga, Filomena Morisco, Ivan Gentile, Giuseppe Portella

**Affiliations:** 1 Departmental Area of Health Services, Unit of Health Services Organization, University Hospital Federico II, Naples, Italy; 2 Department of Translational Medical Sciences, Univeristy of Naples Federico II, Naples, Italy; 3 Department of Clinical Medicine and Surgery, Section of Infectious Diseases, University of Naples Federico II, Naples, Italy; 4 Department of Clinical Medicine and Surgery, Operational Unit of Liver and Biliary Diseases, University Hospital Federico II, Naples, Italy

**Keywords:** HCV, Opportunistic screening, Over 55, Unrecognized infection, HCV antigen, Reflex testing

## Abstract

**Background:**

Hepatitis C virus (HCV) remains a global public health concern. Despite the availability of direct-acting antivirals (DAAs), many infections remain undiagnosed—particularly in older adults excluded from national screening programs. This study aimed to assess the prevalence of unrecognized HCV infections in surgical patients over 55 years of age through an opportunistic hospital-based screening approach.

**Methods:**

A single-center, retrospective observational study was conducted at “Federico II” University Hospital in Naples, Italy, from January 1 to December 31, 2023. All patients aged ≥ 18 years undergoing surgery or hospital admission were screened for anti-HCV antibodies. Reflex testing of samples with a signal-to-cut off (s/co) ratio ≥ 11 was performed with Elecsys^®^ HCV Duo assay to confirm active infection via antigen detection. The primary endpoint was the prevalence of undiagnosed active infections in patients over 55. Secondary endpoints included distribution by age, sex, and hospital department.

**Results:**

Of 33,658 patients screened, 768 (2.3%) tested positive for anti-HCV antibodies; 178 (23.2%) were antigen-positive. Active infections were most common in patients aged 55–79 (66%) and ≥ 80 (21.7%), with the highest positivity (36.5%) in those over 80. In non–high-risk departments, HCV antigen positivity reached 21.2%. No significant sex differences were observed. All antigen-positive patients received DAA treatment.

**Conclusions:**

Opportunistic screening in surgical patients over 55 is effective, feasible, and cost-efficient. The high rate of undiagnosed infections in this age group supports expanding screening efforts to include older adults. These results support the national implementation of opportunistic screening programs targeting older adults to achieve HCV elimination goals. Further multicenter studies are needed to support broader implementation.

**Table:** 

Text box 1. Contributions to literature
• This study addresses a major gap in current hepatitis C screening policies by focusing on adults over 55 years of age, a population with high historical exposure that is largely excluded from national screening programs.
• It provides real-world evidence that hospital-based opportunistic screening can effectively identify previously undiagnosed HCV infections outside traditional “high-risk” clinical settings.
• The study highlights reflex HCV antigen testing as a practical solution to overcome diagnostic delays and loss to follow-up in hospitalized patients.
• These findings support expanding age-based screening strategies to strengthen equity, improve case detection, and accelerate progress toward HCV elimination goals.

## Introduction

Hepatitis C virus (HCV) infection represents a significant global public health issue [1]. According to estimates from the World Health Organization (WHO), over 58 million people live with chronic HCV infection, with approximately 1.5 million new cases diagnosed annually [2]. The infection, often asymptomatic for long periods, is associated with a high risk of progression to chronic liver disease, cirrhosis, and hepatocellular carcinoma, and is among the leading causes of infectious disease-related deaths in middle- and high-income countries surpassing deaths due to HIV and tuberculosis [3, 4]. In fact, in the past two decades, 48% of global hepatitis-related mortality has been attributed to HCV infection [3].

In Italy, it is estimated that approximately 280,000 individuals live with potentially asymptomatic active chronic HCV infection, in addition to about 120,000 individuals with hepatic fibrosis or advanced cirrhosis affected by unresolved HCV infection [5]. Particularly, individuals born before 1969 show a higher incidence, which is attributable to historical risk factors and the previous lack of screening and monitoring protocols [6].

In 2016, WHO set the goal of eradicating HCV by 2030 through integrated strategies of prevention, early diagnosis, and treatment with direct-acting antivirals (DAAs) [7]. One of the fundamental tools for achieving this objective is screening, which enables the identification of infected individuals and their timely initiation into effective therapeutic pathways [8]. To achieve this goal, it is essential that at least 90% of infected individuals be diagnosed, and that 80% of them undergo treatment [7]. However, the large proportion of undiagnosed infections—estimated at approximately 300,000 in Italy—poses a significant barrier to reaching this target [6, 9]. In response to this evidence, and to identify hidden infections, the Italian government revised the target population for free HCV screening, launching a national program aimed at individuals born between 1969 and 1989, inmates, and those enrolled in addiction treatment services [10, 11]. This strategy, however, has certain limitations—particularly the exclusion of older age groups, which show higher prevalence due to more relevant diffusion of the infection in the past decades in absence of adequate measures of prevention [12].

However, older cohorts—particularly individuals born before 1969—are often excluded from screening programs based on the assumption that their lower life expectancy reduces the cost-effectiveness of such interventions. This perspective overlooks substantial epidemiological evidence indicating that these individuals have among the highest rates of undiagnosed active infection, largely due to past exposure to unsafe medical practices and the historical absence of effective prevention measures [6, 12, 13]. Excluding them may therefore undermine national efforts to achieve HCV elimination by 2030, perpetuating a significant “epidemic tail” in the population.

Comparable epidemiological evidence from neighboring Mediterranean countries further supports the need to expand screening efforts beyond currently targeted birth cohorts. In Spain, nationwide seroprevalence studies conducted between 2017 and 2018 reported a high prevalence of active HCV infection among individuals aged 45–64 years, as well as a substantial residual burden in older age groups, reflecting historical patterns of transmission similar to those observed in Italy [14]. Moreover, recent opportunistic population-based programs, such as the CRIVALVIR-FOCUS initiative, have demonstrated that screening older adults remains an effective strategy for identifying unrecognized infections, thereby contributing meaningfully to national elimination goals [15]. These findings reinforce the rationale for extending opportunistic screening to individuals over 55 years of age, who represent a population with both increased historical exposure and persistent diagnostic gaps.

Moreover, conventional diagnostic algorithms, which rely on sequential anti-HCV antibody testing followed by confirmatory HCV RNA testing on a second sample, often face logistical obstacles—particularly in hospital settings with short stays or limited patient availability or with limited resources.

To overcome this bottleneck, our approach incorporated reflex testing for HCV core antigen on the same initial sample, streamlining the diagnostic pathway and improving the likelihood of detecting active infections in a timely manner.

## Methods

### Study design

This was a single-center, retrospective, observational, non-interventional study conducted at the “Federico II” University Hospital in Naples, Italy. The observation period spanned twelve months, from January 1 to December 31, 2023.

### Ethical considerations

The study protocol and informed consent forms were approved by the Campania 3 Ethics Committee. All procedures were conducted in accordance with the Declaration of Helsinki (Fortaleza 2013 revision) and the principles of Good Clinical Practice (ICH-GCP). Data were anonymized and managed in full compliance with the applicable data protection legislation in accordance with the EU General Data Protection Regulation [Regulation (EU) 2016/679].

### Study population

The study included all patients aged 18 years or older who, during the observation period, underwent surgical procedures, diagnostic or therapeutic invasive procedures, or accessing clinical areas with deliberate biological risk exposure at the Federico II University Hospital.

### Screening procedure

During hospitalization, after obtaining informed consent, demographic data including sex and age were collected. All admitted patients were screened for anti-HCV antibodies using the ADVIA Centaur HCV assay (Siemens Healthineers), a two-wash indirect sandwich immunoassay. The assay expresses results as a signal-to-cut-off (s/co) ratio, with values ≥ 1.0 considered positive for anti-HCV antibodies.

However, s/co ratios ≤ 11 are typically indicative of previous, resolved HCV infections or low-level reactivity due to waning antibodies. Conversely, an s/co ratio ≥ 11 is associated with a high likelihood of active HCV infection and was therefore chosen as the threshold for further testing. This threshold is supported by literature showing that only s/co values above 11 provide high specificity and significantly reduce the incidence of false positives, thus improving the accuracy of subsequent diagnostic evaluations [16–19].

Samples with an s/co ratio ≥ 11 were further analysed using the Elecsys^®^ HCV Duo assay (Roche Diagnostics) on the cobas^®^ e 801 platform. This assay enables the simultaneous detection of HCV core antigen and anti-HCV antibodies through two separate outputs: a semi-quantitative signal-to-cut-off ratio for total anti-HCV antibodies, and a qualitative (reactive/non-reactive) result for the HCV core antigen. Sera with an s/co ratio ≥ 11 were thus analysed using the Elecsys^®^ HCV Duo assay for the simultaneous detection of both HCV core antigen and anti-HCV antibodies, with two separated results reported: anti-HCV antibodies (Total, s/co) and HCV core antigen (qualitative result: reactive/non-reactive).

The analytical system automatically calculates results by comparing the sample signal to a calibration curve generated with internal calibrators. The antibody result is expressed as a signal-to-cut-off (s/co) ratio, providing a semi-quantitative indication of antibody response intensity, while the antigen test provides a qualitative result.

The decision to implement reflex HCV antigen testing instead of conventional HCV RNA detection was based on both logistical and diagnostic considerations. In hospital settings, the need for an additional blood draw after anti-HCV positivity often results in incomplete diagnostic pathways due to early patient discharge or limited availability. By using the same sample for reflex testing, this workflow minimizes patient loss and expedites diagnosis. Furthermore, existing literature has demonstrated that in patients with an anti-HCV s/co ratio ≥ 11, the positive predictive value of HCV antigen detection for identifying active infection approaches 95%, making it a reliable and cost-effective alternative to RNA testing in real-world scenarios [16–19].

### Study objectives

The primary endpoint was to evaluate the prevalence of HCV infection in patients, with specifc attention to subjects with more than 55 years of age—individuals excluded from national screening programs.

The secondary endpoint was to analyse the distribution of active, HCV infection by age group (≥ 18 - ≤33 years; ≥34 - ≤54 years; ≥55 - ≤79 years; ≥80 years), sex, and type of hospital department. Departments were classified as “high-risk” if they included Gastroenterology, Hepatology, or Infectious Diseases units, given the higher probability of HCV infection documented in literature in patients observed in these clinical setting [20].

### Sample size

The sample size was not calculated a priori, as this was a convenience sample study based on consecutive patient inclusion. All eligible individuals who accessed the ‘Federico II’ Academic Hospital between January 1 and December 31, 2023, were included, for a total of 33,658 participants.

### Statistical analysis

Statistical analyses were conducted using SPSS version 23.0 (IBM Corporation, Armonk, NY, USA). The normality of variable distributions was assessed using the Kolmogorov-Smirnov test. Continuous variables with a normal distribution were expressed as means ± standard deviations (SD). Categorical variables were described using frequencies and percentages. Group differences were analyzed using the chi-square test or Fisher’s exact test, as appropriate. Analyses were stratified by age group, sex, and department type (high-risk, i.e., Gastroenterology, Hepatology, Infectious Diseases vs. general units). Statistical significance was set at *p* ≤ 0.05 for all tests.

## Results

Among the 33,658 patients analysed between January 1 and December 31, 2023, none declined participation. Of these, 768 on 33,658 individuals (2.3%), with a mean age of 69.4 ± 12.8 years and 58.5% being male, tested positive for anti-HCV antibodies with an s/co ratio ≥ 11.

The majority of these patients were in the 55–79 age group (66.0%), followed by those aged ≥ 80 years (21.7%), 34–54 years (11.5%), and 18–33 years (0.8%) (Table 1). These proportions describe the age distribution within the anti-HCV–positive cohort and should not be interpreted as age-specific prevalence in the entire screened population.

**Table 1:** Demographic characteristics of anti-HCV antibody positive patients (s/co ≥11)

Age Group	Anti-HCV Positive (s/co ≥11) *n*, %	Mean Age (±SD)	Male Sex *n*, %
≥18 – ≤33 years	6, 0.8%	28.3 (±3.3)	3, 50%
≥34 – ≤54 years	88, 11.5%	45.8 (±5.4)	57, 64.8%
≥55 – ≤79 years	507, 66%	69.1 (±7.3)	299, 59%
≥80 years	167, 21.7%	83.9 (±3.1)	90, 53.9%
Total	768, 100%	69.4 (±12.8)	449, 58.5%

Percentages represent the distribution of anti-HCV–positive individuals within the anti-HCV–positive cohort (*n* = 768) and should not be interpreted as age-specific prevalence in the entire screened population

All antibody-positive samples were further tested using the HCV DUO assay on the same aliquot, confirming the presence of HCV antigen in 178 patients (23.2%), and indicating active infection. Among them, 42.1% were female and 57.9% were male, with no statistically significant difference between sexes.

Age-stratified analysis showed significant variation in antigen positivity across age groups. Among patients aged ≥ 80 years, 36.5% tested positive for HCV antigen. This was followed by 21.6% in the 34–54 age group, 19.1% in the 55–79 group, and 16.7% in the 18–33 group. These percentages represent the distribution of HCV antigen–positive cases within the anti-HCV–positive subgroup, rather than the prevalence of active infection in each age group among all screened individuals. When considering the distribution of antigen-positive cases across the entire cohort, most active infections occurred in patients aged > 55 years, particularly those in the 55–79 and ≥ 80 age groups with a statistically significant association between increasing age and antigen presence (Table 2).

**Table 2:** Distribution of HCV antigen–positive cases within the anti-HCV–positive population, stratified by age group

Age Group	Anti-HCV Positive (s/co ≥11) *n*, %	HCV-Ag Positive *n*, %
≥18 – ≤33 years	6, 0.8%	1, 16.7%
≥34 – ≤54 years	88, 11.5%	19, 21.6%
≥55 – ≤79 years	507, 66%	97, 19.1%
≥80 years	167, 21.7%	61, 36.5%
Total	768, 100%	178, 23.2%

Percentages represent the proportion of HCV antigen–positive individuals within each age subgroup of anti-HCV–positive patients

When stratified by hospital department, patients identified in “high-risk” units (i.e., Gastroenterology, Hepatology, Infectious Diseases) exhibited a 2.1% HCV antigen positivity rate, compared to 21.2% in patients from other departments and outpatient clinics. This difference was not statistically significant.

A further analysis was conducted correlating age groups with high-risk departments. Sixteen patients (20.5%) from these departments tested positive for HCV antigen. Among these, the highest proportion was in the 55–79 age group (14.1% of the total), followed by those aged ≥ 80 years (5.1%). The 34–54 and 18–33 age groups showed low or no prevalence (1.3% and 0%, respectively). Within age strata, antigen positivity was 6.3% in the 34–54 group, 22.9% in the 55–79 group, and 21.1% in those over 80, with no positive cases among those aged 18–33. Although not statistically significant, there was a slight trend toward higher positivity in older age groups within high-risk departments.

All patients who tested positive for HCV antigen were subsequently managed according to the standard follow-up protocols established for HCV-infected individuals, ensuring appropriate linkage to care and treatment in line with regional clinical guidelines.

## Discussion

This study demonstrated that opportunistic screening for HCV infection in surgical patients over the age of 55 allows for the identification of a significant number of previously unrecognized infections. Although this population is at high risk, it is currently excluded from national screening programs, which primarily target individuals born between 1969 and 1989. The proposed approach was shown to be logistically and economically sustainable, well tolerated, and potentially effective in contributing to HCV elimination by 2030, in alignment with the objectives set by the World Health Organization [7].

Recent data from Italy revealed that the prevalence of anti-HCV antibodies in hospitalized patients over the age of 76 can exceed 5%, significantly higher than in the 1969–1989 cohort, reinforcing the need to extend screening efforts to older population groups [13].

These findings align with other studies indicating that in-hospital screening programs report an overall prevalence of 3.03% among patients admitted for non-HCV-related conditions, with even higher rates in older age groups [13].

Diagnosing HCV infection in older age groups is crucial, as these individuals often require frequent contact with the healthcare system, particularly during hospitalization or assisted care. In such settings, there is a risk of HCV transmission, which may contribute to the further spread of the virus [13, 20]. Notably, during the study, an elderly patient was diagnosed with active HCV infection prior to undergoing surgery. Following the procedure, the operating surgeon experienced a needlestick injury and subsequently contracted the infection, as confirmed by a positive HCV RNA test.

HCV infection often remains asymptomatic for decades, delaying diagnosis until the onset of severe complications such as cirrhosis or hepatocellular carcinoma [21]. In our sample, 23.2% of anti-HCV-positive individuals were found to have active infection, with a peak of 36.5% among those aged 80 years or older. These results support the notion that current age-based screening criteria are overly restrictive and contribute to a persistent reservoir of undiagnosed infections. Moreover, the distribution of HCV-Ag positive cases in so-called “low-risk” departments—with a positivity rate of 21.2% among anti-HCV-positive patients—suggests that infections can be detected across diverse clinical settings, in a relavant percentage, even in the absence of hepatic symptoms or known comorbidities. Extending screening to patients over 55 could substantially increase diagnostic rates. This is particularly justified considering that the prevalence of active HCV infection (HCV RNA-positive) reaches 32.6% among individuals born between 1947 and 1968, and up to 44.4% in the 1969–1989 cohort [13].

A recent retrospective study in an Italian tertiary hospital found that the prevalence of anti-HCV antibodies among patients over 76 years old was 5.3%, nearly triple that observed in the 1969–1989 birth cohort (1.5%). Furthermore, only a small portion of antibody-positive individuals underwent confirmatory HCV-RNA testing, highlighting the urgent need for more inclusive and proactive screening strategies [13].

These findings are consistent with recent evidence showing critical gaps in hospital-based HCV screening programs, particularly regarding the lack of attention to comorbidities and socioeconomic disparities, both of which are key determinants in access to diagnostics and care [22].

While our study did not focus on hepatic comorbidities, recent research has highlighted the value of opportunistic screening even in non-infectious, non-hepatologic contexts. For example, Weiss et al. showed that assessing hepatic steatosis during lung cancer screening has prognostic value, reinforcing the importance of integrated evaluations in hospital settings [23].

The combined use of serological and HCV-Duo testing in our study enabled immediate detection of active viral antigen, enhancing both diagnostic speed and reliability. The screening process proved simple, minimally invasive, and easily integrated into existing clinical pathways.

The diagnostic strategy adopted in this study deviates from the standard recommended algorithm, which is based on initial anti-HCV antibody testing followed by confirmatory HCV RNA detection.

Although current American Association for the Study of Liver Diseases; Infectious Diseases Society of America (AASLD–IDSA) guidance recommends performing both anti-HCV antibody testing and confirmatory HCV RNA (or antigen) testing on the same initial blood sample—thus eliminating the need for an additional venipuncture—real-world implementation of this workflow is often limited by organizational and logistical barriers. In many hospital settings, rapid patient turnover or incomplete laboratory pathways may prevent confirmatory RNA testing from being performed on the same specimen, resulting in missed diagnoses. Our reflex antigen strategy was specifically designed to overcome these operational bottlenecks and ensure uninterrupted diagnostic progression. This approach is consistent with the recommendations outlined in the AASLD–IDSA HCV Guidance, which explicitly endorses reflex confirmatory testing using the same sample to facilitate timely diagnosis and linkage to care [24]. As a result, the diagnostic process remains incomplete in a substantial number of cases. To overcome this limitation, we implemented reflex testing for HCV core antigen (HCV Ag) on the same sample originally collected for anti-HCV screening. This enabled uninterrupted progression of the diagnostic workflow within the existing specimen pathway.

It is important to note that we used the HCV DUO assay by Roche Diagnostics for reflex detection of HCV antigen. However, this is a combination assay that enables the simultaneous detection of both anti-HCV antibodies and HCV antigen. Therefore, in laboratories equipped with Roche instruments, it may be possible to achieve even faster identification of active HCV infections by testing samples directly with the HCV DUO assay alone.

Notably, we have previously demonstrated the utility of reflex testing in enhancing viral hepatitis diagnostics: reflex anti-HDV testing significantly increased the detection rate of hepatitis D virus (HDV) infections by identifying previously unrecognized cases [25].

Organizational models of hospital screening have already been implemented in other Italian regions, successfully identifying unrecognized active infections, contributing to micro-elimination strategies [13]. To confirm active HCV infection among patients testing positive for anti-HCV antibodies, HCV RNA testing is required. However, in a referenced cohort, only a small fraction of seropositive individuals underwent confirmatory testing: among 1,297 patients who tested anti-HCV positive, merely 198 (15.3%) were assessed for the presence of HCV RNA [13].

By contrast, in our study, all patients with anti-HCV positivity and a high pre-test probability of active infection were systematically tested using a reflex HCV Ag approach, thereby demonstrating the operational efficiency and clinical utility of this strategy.

Comparable limitations have been noted in another hospital-based screening program, where the HCV RNA testing rate reached only 38% among anti-HCV positive individuals. In that setting, a substantial proportion of missed confirmatory tests was attributed to patient discharge prior to RNA testing, ultimately compromising the effectiveness of the screening protocol [26].

These findings underscore a broader structural issue observed across hospital-based HCV screening initiatives: the low rate of confirmatory viremia testing among anti-HCV–positive patients is largely driven by logistical delays rather than clinical factors. As the Reviewer correctly highlights, this gap has significant implications for timely diagnosis and linkage to care. Implementing universal reflex testing—either through HCV RNA or HCV core antigen—on all anti-HCV–positive samples represents a crucial strategy to prevent missed diagnoses, reduce healthcare delays, and ensure rapid therapeutic referral. Reflex testing eliminates dependence on patient availability for additional blood sampling and minimizes workflow interruptions, thereby aligning hospital practice with international recommendations and supporting efficient HCV elimination pathways.

Identifying patients eligible for DAA therapy facilitates timely access to treatments with > 95% efficacy, while also reducing the risk of nosocomial transmission and the clinical and economic burden of advanced liver disease. The success of such screening initiatives depends heavily on ensuring rapid linkage to specialist care and treatment, with integrated hospital models achieving linkage-to-care rates above 70% [27].

All patients identified with active infection were referred to hepatology services and initiated on DAA therapy. Most received pangenotypic regimens such as sofosbuvir/velpatasvir or glecaprevir/pibrentasvir, which are recommended for older adults due to their high efficacy, limited drug–drug interactions, and favorable tolerability profile. Available follow-up data indicated excellent adherence and virological clearance in the vast majority of treated individuals, in line with real-world evidence showing SVR rates above 95% even in elderly and comorbid populations.

The opportunistic approach adopted in this study emerges as a key strength. It allows for the identification of patients with previously unrecognized infections who might otherwise not have been referred for timely treatment. Our findings reinforce the need to structurally embed reflex antigen or RNA testing in hospital settings, as the absence of reflex confirmation contributes to diagnostic delays and undermines micro-elimination strategies. In addition, compared to commonly used rapid tests, the laboratory-based methods employed offered superior sensitivity and specificity, improving diagnostic accuracy and minimizing false negatives. However, implementing hospital-based screening protocols may face organizational challenges, such as staff workload, patient consent logistics, and integration into existing laboratory workflows. To overcome these barriers, streamlined procedures including pre-admission consent, automated reflex testing orders, and training for clinical staff are recommended. Embedding screening in routine care pathways—such as surgical admissions or day-hospital procedures—can further enhance feasibility without adding significant operational burden. One of the main advantages of our approach is the use of a cost-effective test compared to Real-Time PCR. In addition, the assay requires minimal technical expertise and is less demanding in terms of time and labor. A cost-effectiveness analysis conducted among Italian patients demonstrated that cohort-based screening for those born between 1948 and 1988, followed by DAA treatment, was highly cost-effective, with an incremental cost-effectiveness ratio of €3,552 per quality-adjusted life year gained [6]. In addition, recent real-world studies have demonstrated that HCV antigen testing is a valid and economically sustainable alternative to HCV RNA, especially in settings where logistical limitations may hinder RNA testing. Compared to nucleic acid amplification tests, HCV antigen assays offer lower costs, faster turnaround times, and reduced need for highly specialized laboratory infrastructure—making them particularly suitable for large-scale hospital-based screening campaigns.

Nevertheless, some limitations of our study must be acknowledged. Its single-center nature may limit generalizability. Further multicenter studies and formal health economic evaluations are necessary to support broader implementation of similar strategies. Another limitation of our study is the absence of age-stratified denominators for the anti-HCV–negative population. For this reason, we report age distributions within the anti-HCV–positive subgroup rather than age-specific prevalence in the entire screened cohort.

In conclusion, the findings of this study reinforce both the clinical and public health rationale for introducing HCV screening in hospitalized patients over the age of 55. Implementing such screening in hospital settings also offers an opportunity to reach populations that are often inaccessible to community-based programs, such as frail elderly individuals and those with limited access to care, who are frequently excluded from national screening algorithms [13]. Opportunistic screening continues to emerge as a promising strategy for early identification of infection, which is essential for achieving the WHO target of eliminating HCV by 2030. From a public health ethics perspective, the systematic exclusion of individuals over 55 from national screening programs raises concerns regarding equity and justice. Older adults represent a population with historically higher exposure to iatrogenic risk factors and limited access to preventive strategies. Failing to address their diagnostic needs not only compromises elimination goals but also reinforces disparities in healthcare access and outcomes. Including this group in targeted screening strategies is essential for promoting fairness and reducing avoidable health inequities.

## Conclusions

The results of this study confirm the effectiveness and sustainability of opportunistic HCV screening in patients over the age of 55, a population segment with a high prevalence of active infection that is currently excluded from national screening programs. The high percentage of HCV antigen-positive individuals, particularly among older age groups and in non-infectious, non-hepatologic setting, highlights the existence of a substantial hidden reservoir of unrecognized infections—potentially transmissible and amenable to effective treatment.

The integration of HCV testing at admission in hospital proved to be logistically simple and compatible with existing clinical workflows, offering a concrete opportunity for identification of infected individuals and timely initiation of DAA treatment. In our study, all patients with active infections identified through screening were promptly referred and treated with DAAs, with the goal of achieving viral eradication and reducing the risk of long-term complications.

The combined use of highly specific serological tests and antigen assays ensured high diagnostic accuracy, minimized the risk of false positives, and optimized resource utilization.

Beyond logistical feasibility, this approach also demonstrated potential economic sustainability, owing to its integration into existing hospital processes and the use of high-specificity laboratory methods that reduce the number of unnecessary or inconclusive tests. These characteristics make screening a scalable and replicable strategy, even in resource-limited settings.

Based on these findings, we propose systematically extending HCV screening to all hospitalized patients over the age of 55, regardless of known risk factors or clinical symptoms. This approach may represent a strategic step toward HCV elimination in Italy, contributing to the reduction of the clinical, social, and economic burden of the disease.

In this perspective, it may also be valuable to assess the implementation of support cohorts—i.e., additional groups to be screened in parallel—to enhance the overall effectiveness of the strategy and intercept further unrecognized cases.

Further multicenter studies, combined with cost-effectiveness analyses, will be necessary to validate and generalize the results and to support the formal inclusion of HCV screening in national hospital protocols.

## Data Availability

The datasets generated and/or analyzed during the current study are not publicly available due to privacy restrictions but are available from the corresponding author on reasonable request.
